# Clinical Relevance and Molecular Pathogenesis of the Emerging Serotypes 22F and 33F of *Streptococcus pneumoniae* in Spain

**DOI:** 10.3389/fmicb.2020.00309

**Published:** 2020-02-27

**Authors:** Julio Sempere, Sara de Miguel, Fernando González-Camacho, José Yuste, Mirian Domenech

**Affiliations:** ^1^Centro Nacional de Microbiología, Instituto de Salud Carlos III, Madrid, Spain; ^2^Servicio de Epidemiología de la Comunidad de Madrid, Dirección General de Salud Pública, Madrid, Spain; ^3^Centro de Investigación Biomédica en Red de Enfermedades Respiratorias (CIBERES), Madrid, Spain

**Keywords:** *Streptococcus pneumoniae*, PCV-pneumococcal conjugate vaccine, biofilms, serotype 22F, serotype 33F, PSGL-1

## Abstract

*Streptococcus pneumoniae* is the main bacterial cause of respiratory infections in children and the elderly worldwide. Serotype replacement is a frequent phenomenon after the introduction of conjugated vaccines, with emerging serotypes 22F and 33F as frequent non-PCV13 serotypes in children and adults in North America and other countries. Characterization of mechanisms involved in evasion of the host immune response by these serotypes is of great importance in public health because they are included in the future conjugated vaccines PCV15 and PCV20. One of the main strategies of *S. pneumoniae* to persistently colonize and causes infection is biofilm formation. In this study, we have evaluated the influence of capsule polysaccharide in biofilm formation and immune evasion by using clinical isolates from different sources and isogenic strains with capsules from prevalent serotypes. Since the introduction of PCV13 in Spain in the year 2010, isolates of serotypes 22F and 33F are rising among risk populations. The predominant circulating genotypes are ST433^22*F*^ and ST717^33*F*^, being CC433 in 22F and CC717 in 33F the main clonal complexes in Spain. The use of clinical isolates of different origin, demonstrated that pediatric isolates of serotypes 22F and 33F formed better biofilms than adult isolates and this was statistically significant. This phenotype was greater in clinical isolates from blood origin compared to those from cerebrospinal fluid, pleural fluid and otitis. Opsonophagocytosis assays showed that serotype 22F and 33F were recognized by the PSGL-1 receptor on leukocytes, although serotype 22F, was more resistant than serotype 33F to phagocytosis killing and more lethal in a mouse sepsis model. Overall, the emergence of additional PCV15 serotypes, especially 22F, could be associated to an enhanced ability to divert the host immune response that markedly increased in a biofilm state. Our findings demonstrate that pediatric isolates of 22F and 33F, that form better biofilm than isolates from adults, could have an advantage to colonize the nasopharynx of children and therefore, be important in carriage and subsequent dissemination to the elderly. The increased ability of serotype 22F to avoid the host immune response, might explain the emergence of this serotype in the last years.

## Introduction

*Streptococcus pneumoniae*, also termed pneumococcus, colonizes asymptomatically the human nasopharynx although the bacterium has the capacity to reach the lower respiratory tract (LRT) producing pneumonia and disseminate producing invasive pneumococcal disease (IPD) (Bogaert et al., 2004). The burden of disease caused by this microorganism is of great relevance because is the major etiologic agent of community acquired pneumonia, non-epidemic meningitis and a frequent cause of bacterial sepsis (Bogaert et al., 2004). As a result, this devastating pathogen is responsible for high morbidity and mortality rates worldwide affecting mainly the children under the age of 5 years and adults older than 65 years. Indeed, nearly 2.38 million deaths are caused by LRT infections, being *S. pneumoniae* the major etiologic agent of these alarming rates (GBD 2016 Lower Respiratory Infections Collaborators, 2018). As a consequence, LRT infections are the sixth mortality cause for all ages and the first cause of death among children under 5 years old (GBD 2016 Lower Respiratory Infections Collaborators, 2018). While the use of available antibiotics is the best option to treat pneumococcal infections once they are established, the emergence of multidrug resistant strains can jeopardize the outcome of the infection in many cases (Aguinagalde et al., 2015; Azarian et al., 2018).

Prophylactic measures based on the administration of pneumococcal vaccines in children and adults, seems to be the best strategy to control the development of IPD and pneumonia and reduce the impact of antimicrobial resistance (Moore et al., 2015; Atkins et al., 2018; Wahl et al., 2018). In children under 2 years old, there are currently two vaccines available, a 10-valent conjugate vaccine (PCV10) and a 13-valent conjugate vaccine (PCV13). The use of PCV10 has decreased the burden of disease caused by serotypes covered in the vaccine but several countries using this vaccine have reported the emergence of non-PCV10 serotypes such as 19A after its introduction (Savulescu et al., 2017; Cassiolato et al., 2018; Rinta-Kokko et al., 2018). PCV13 has shown to be effective in children who were the main target for this vaccine but also has shown herd effects for the elderly population (Mackenzie et al., 2016). In adults, the 23-valent polysaccharide vaccine (PPV23) can be used to prevent IPD although its effectiveness against pneumonia is controversial (Huss et al., 2009; Andrews et al., 2012; Russell et al., 2015; Tin Tin Htar et al., 2017). The use of PCV13 has been shown to be effective avoiding IPD and pneumonia in the elderly population (Bonten et al., 2015; McLaughlin et al., 2018). The main limitation of these vaccines is that serotype replacement is a frequent phenomenon that occurs after the massive use of these vaccines based on capsular polysaccharides due to the wide antigenic variability of this bacterium with up to 99 serotypes described so far and emergence of virulent clones with vaccine-escape capsule (Brueggemann et al., 2007; Domenech et al., 2018). To target the emergence of non-vaccine types producing pneumococcal infection, a 15-valent conjugate vaccine (PCV15) including serotypes covered by PCV13 plus serotypes 22F and 33F is in clinical phase III with the advantage of inducing a higher title of antibodies against serotype 3 (Greenberg et al., 2018; Stacey et al., 2019). In addition, a 20-valent conjugate vaccine (PPV20) that contains serotypes in PCV15 plus serotypes 8, 10A, 12F, 11A, and 15B is also in clinical trials studies (Thompson et al., 2019).

The emergence of many of these serotypes in certain countries after the use of current PCVs is one of the main reasons for including these additional serotypes in newer conjugate vaccines. In United States, serotypes 22F and 33F were the main cause of IPD among non-PCV13 in children under 5 years old and adults ≥65 years (Moore et al., 2015). In Europe, several countries have shown the rise of non-PCV13 serotypes in children and adults including serotypes covered by PCV15 and PCV20 (Aguinagalde et al., 2015; Savulescu et al., 2017; Ladhani et al., 2018). Nevertheless, one aspect that is poorly investigated before the introduction of new conjugate vaccines is the pathogenic capacity of these additional serotypes.

It is been known that biofilm formation in *S. pneumoniae* provides the bacterium an advantage in terms of virulence because it increases the resistance to antimicrobial drugs and enhances the ability to avoid the host immune response (Donlan and Costerton, 2002; Domenech et al., 2013). For instance, serotypes 11A and 35B, which are serotypes frequently associated to multidrug resistance, are good biofilm formers (Domenech et al., 2015). Biofilm formation is a critical aspect from the pathogenesis perspective because *S. pneumoniae* has been reported to form biofilms in the upper respiratory tract (Wu et al., 2017; Iverson et al., 2019; Silva and Sillankorva, 2019) and nasopharyngeal colonization is the initial step in this process and the prerequisite for developing IPD (Bogaert et al., 2004). Interestingly, active infection is also associated to pneumococcal biofilms in meningitis by binding to brain microvascular endothelial cells and in persistent respiratory infection affecting patients with cystic fibrosis or chronic obstructive pulmonary disease (COPD) (Orihuela et al., 2009; Vidal et al., 2013; Vandevelde et al., 2014; Dennis et al., 2018). In the biofilm state, pneumococcal cells express the PspC protein in a higher extent, and this protein is the bacterial ligand associated to the recruitment of factor H and therefore to the cleavage of the C3 complement system protein deposited on the bacterial surface (Domenech et al., 2013). This is interesting in terms of virulence because when pneumococcal cells are released from the biofilm producing dissemination to the LRT, the bloodstream or crossing the blood brain barrier producing meningitis, these colonies express higher levels of the virulence factor PspC increasing the potential for IPD (Yuste et al., 2010).

In contrast to *Neisseria meningitidis*, bactericidal activity by the membrane attack complex of the complement system plays a minor role against *S. pneumoniae* and killing by professional phagocytes in the presence or not of specific antibodies, is the most efficacy mechanism to fight the pneumococcal infection (Standish and Weiser, 2009). For this reason opsonophagocytosis killing assays (OPKA) are the best parameter to measure the functional opsonic activity of antibodies to *S. pneumoniae* when a new vaccine is tested (Greenberg et al., 2018; Stacey et al., 2019; Thompson et al., 2019). Hence, investigating these aspects will contribute to increase the knowledge about the potential of non-vaccine serotypes to produce infection in the host. Differences in the recognition of pneumococcal serotypes by specific components of the host immune response may explain the prevalence of certain serotypes causing IPD (Hyams et al., 2011). In this sense, the human P-selectin glycoprotein ligand 1 is a novel receptor for *S. pneumoniae* that contributes to protection against IPD by controlling the severity of the infection (Ramos-Sevillano et al., 2016). This receptor is present on leukocytes that recognizes the pneumococcal capsular polysaccharide and the main autolytic enzyme of the bacterium (the amidase LytA) increasing the phagocytosis process (Ramos-Sevillano et al., 2016). Differences in the interaction of different pneumococcal capsules with PSGL-1 might explain the higher burden of disease caused by certain serotypes of *S. pneumoniae* (Ramos-Sevillano et al., 2016).

In this study, we have evaluated the impact of current vaccine strategies in the epidemiology of serotypes 22F and 33F during the period 2009–2018 including the circulating genotypes that cause IPD among these serotypes. In addition, we have characterized the ability of serotypes 22F and 33F to form biofilms and their capacity to avoid the phagocytosis process and produce infection because they are important aspects that are missing for these additional serotypes covered in the new PCV15. For comparison purposes, we also included pneumococcal serotypes that are prevalent in IPD and/or because they are associated to multidrug resistance.

## Materials and Methods

### Strains, Media and Genetic Transformation

The pneumococcal strains of the different serotypes used in this study are listed in Table 1. The Spanish Pneumococcal Reference Lab (SPRL) receives the IPD cases from hospitals located at all the Spanish regions through a passive surveillance system with minimal required information accompanying isolates (hospital and city, sample, age of the patient and date of isolation). The SPRL notifies annually to the European Center for Disease Control (ECDC) all the IPD cases received following a passive surveillance system that cover 80% of the national level according to estimates by the National Center for Epidemiology reported to ECDC (ECDC, 2018). Unfortunately, vaccine coverage data is not available and that is the reason that effectiveness of the different vaccines cannot be included in this study. Initially, PCV7 coverage based on vaccine sales was low, but its use increased from 2002 onward with reported vaccine coverage below 50% before 2006 and missing data on PCV coverage at national level in further years (Fenoll et al., 2015).

**TABLE 1:** Strains of *S. pneumoniae* used in this study^a^.

**Strain**	**Description/serotype (source)b ST/CCc**	**References/sourced**
M11	Non-encapsulated strain derived from R6 (Hex^–^, lytA^+^)	Domenech et al., 2014
YNM2	M11 transformant with DNA from strain 1734/19; serotype 8	This study
1734/19	8(blood)	SPRL
YNM3	M11 transformant with DNA from strain 1732/19; serotype 11A	This study
1732/19	11A(blood)	SPRL
YNM4	M11 transformant with DNA from strain 1228/19; serotype 19A	This study
1228/19	19A(blood)	SPRL
P007	M11 transformant with DNA from strain 406; serotype 3	Domenech et al., 2009
P224	M11 transformant with DNA from strain 3017/13; serotype 24F	Domenech et al., 2014
P244	M11 transformant with DNA from strain 3014/13; serotype 22F	Domenech et al., 2015
1407/18	22F(blood/pediatric) ST3134/CC1439	SPRL
1000/18	22F(blood/adult) ST13692/CC698	SPRL
1155/18	22F(CSF/pediatric) ST433/CC433	SPRL
523/18	22F(CSF/adult) ST13692/CC698	SPRL
223/11	22F(PF/pediatric) ST433/CC433	SPRL
1285/18	22F(PF/adult) ST698/CC698	SPRL
2780/17	22F(Otic/adult) ST13692/CC698	SPRL
100/08	22F(Otic/pediatric) ST433/CC433	SPRL
1766/18	22F(blood/pediatric) ST3134/CC1439	SPRL
2597/17	22F(blood/pediatric) ST433/CC433	SPRL
194/17	22F(blood/pediatric) ST433/CC433	SPRL
2153/17	22F(blood/pediatric) ST433/CC433	SPRL
212/18	22F(blood/adult) ST698/CC698	SPRL
250/18	22F(blood/adult) ST433/CC433	SPRL
306/18	22F(blood/adult) ST433/CC433	SPRL
613/18	22F(blood/adult) ST7314/CC433	SPRL
P017	M11 transformant with DNA from strain SSISP33F/1; serotype 33F	Domenech et al., 2015
1833/18	33F(blood/pediatric) ST717/CC717	SPRL
1950/18	33F(blood/adult) ST13320/CC717	SPRL
1088/16	33F(CSF/pediatric) ST717/CC717	SPRL
934/19	33F(CSF/adult) ST717/CC717	SPRL
800/18	33F(PF/pediatric) ST717/CC717	SPRL
363/18	33F(PF/adult) ST717/CC717	SPRL
782/15	33F(Otic/pediatric) ST1012/CC1012	SPRL
1945/15	33F(Otic/pediatric) ST717/CC717	SPRL
644/18	33F(blood/pediatric) ST717/CC717	SPRL
1018/18	33F(blood/pediatric) ST717/CC717	SPRL
1992/18	33F(blood/pediatric) ST4668/CC717	SPRL
2316/17	33F(blood/pediatric) ST13320/CC717	SPRL
1897/18	33F(blood/adult) ST717/CC717	SPRL
2027/18	33F(blood/adult) ST717/CC717	SPRL
627/18	33F(blood/adult) ST717/CC717	SPRL
840/18	33F(blood/adult) ST717/CC717	SPRL

*^*a*^Strains are organized by serotype. ^*b*^CSF, cerebrospinal fluid; PF, pleural fluid. ^*c*^ST, sequence type; CC, clonal complex. ^*d*^SPRL, Spanish Pneumococcal Reference Laboratory.*

All pneumococcal strains were grown in C medium supplemented (C+Y medium) or in Mueller Hinton agar supplemented with 5% defibrinated sheep blood (Becton Dickinson GmbH; Heidelberg, Germany). Bacterial growth was monitored by measuring the absorbance at 550 nm (*A*_550_). *S. pneumoniae* was transformed with chromosomal DNA by treating pre-competent cells with 100 ng/ml of synthetic competence-stimulating pheromone 1 at 37°C for 10 min to induce competence, followed by incubation at 30°C during DNA uptake. Encapsulated transformants of strain M11 were enriched by successive transfers of the transformed culture to C medium containing 0.08% bovine serum albumin, and supplemented with 0.5 μl/ml of anti-R antiserum before plating (Mollerach et al., 1998; Domenech et al., 2015). Anti-R (antisomatic) antiserum contains group-specific agglutinins, which, at the proper dilution, agglutinate only non-encapsulated pneumococci. In this study, we have used isogenic mutants in CPS in order to reduce the influence of genetic variability within the strains. To confirm the absence of mutations in other parts of the genome, we used two independent transformants of each serotype with not differences in the phenotype. Based on this, only one transformed strain of each serotype was further studied for the rest of the experiments. The selection of pneumococcal isolates for immune response characterization and biofilm formation was randomly chosen. We selected five clinical isolates from each source (20 clinical isolates of serotype 22F and another 20 clinical isolates of serotype 33F) from different geographical locations in Spain (Table 1). Moreover, we did biofilm formation trials in parallel with clinical strains of the serotypes studied as control.

### *S. pneumoniae* Typification

In addition to strains showed in Table 1, we included the clinical isolates of serotypes 22F and 33F received at the Spanish Pneumococcal Reference Laboratory (SPRL) since 2009 to analyze the evolution of IPD caused by both serotypes in Spain. We considered IPD clinical isolates those isolated from sterile sites. Serotyping was performed by Quellung reaction, dot blot assay (Fenoll et al., 2015) using specific antisera from the Statens Serum Institut (Copenhagen, Denmark) and/or by molecular capsular sequence typing methodology (Elberse et al., 2011).

Multilocus sequence typing (MLST) was determined with the tools provided by the MLST website^1^. Data were analyzed using Lasergene^®^SeqMan Pro (DNAStar, Madison, WI, United States). Sequence types (STs) were identified based on the allelic profiles of seven housekeeping genes (*aroE*, *ddl*, *gdh*, *gki*, *recP*, *spi*, and *xpt*). We used the eBURST tool to determine the clonal complex (CC) (Feil et al., 2004). STs were considered from the same CC if they demonstrated ≥5 MLST allele numbers in common.

### Biofilm Formation Assay

Biofilm formation assays were performed following the methodology previously described (Domenech et al., 2015) and it was determined by the ability of cells to adhere to the walls and base of 96-well, flat-bottomed polystyrene microtiter dishes (Falcon 353072, Corning Incorporated, New York, NY, United States). Unless stated otherwise, cells were grown in C+Y medium to an *A*_550_ of ≈0.5–0.6, sedimented by centrifugation, resuspended in an equal volume of the indicated pre-warmed medium, diluted 1/100, and then dispensed 200 μl per well. After 5 h of incubation at 34°C, the *A*_595_ was determined using Tecan Infinite F200 (Tecan Group Ltd., Switzerland). The biofilm formed was stained with 0.2% crystal violet (Sigma-Aldrich) and rinsed three times with distilled water to remove non-adherent bacteria. Biofilm formation was quantified solubilizing the biofilm in 95% ethanol (200 μl per well) and measuring the *A*_595_.

### Phagocytosis of *S. pneumoniae* Biofilms and Planktonic Cultures

Experiments investigating human neutrophil phagocytosis were performed by OPKA using HL-60 cells (CCL-240; ATCC) differentiated to granulocytes (Domenech et al., 2013). The presence of complement receptors on HL-60 granulocytes has been previously documented and therefore expression of CD11b (iC3b receptor and CR3 α-chain), a marker of granulocytic differentiation, was measured prior to phagocytic assays to confirm the presence of the receptor (Ramos-Sevillano et al., 2016). After the incubation process at 34°C (previously described in biofilm formation), bacterial cultures growing as biofilms in microtiter plates or the planktonic cultures in tubes were washed with fresh C medium and resuspended in Hank’s buffered salt solution (HBSS) in the presence of calcium and magnesium ions. Biofilm disaggregation was performed by gently pipetting and slow vortexing before the opsonization process to avoid possible bias by morphological differences between the two growing stages. The number of biofilm-forming CFU was determined by viable counts of bacteria and a similar number of planktonic cells were used in each phagocytosis assay. OPKA were performed using 10^5^ HL-60 cells and 2.5 × 10^2^ CFU of *S. pneumoniae* strains (MOI of 400 HL-60:1 *S. pneumoniae*) that were previously opsonized for 1 h with 1/20 of rabbit serum in the case of planktonic cultures, and 20 min with 1/5 of rabbit serum in the case of biofilm cultures. First, the corresponding bacterial suspensions (10 μl) were added to microtiter round bottom polystyrene plates (Nunclon Delta Surface; Themo Fisher Scientific) containing 10 μl of rabbit serum diluted 1/20 or 1/5 in phosphate-buffered saline (PBS, pH 7.0) previously described (Domenech et al., 2013). After incubation at 37°C with shaking (150 rpm) to allow opsonization by the different serum components, HL-60 cells were added and the plate was incubated at 37°C with shaking (150 rpm) for 45 min. The mixture was plated on blood agar plates for bacterial counts determination and results were expressed as bacterial killing. Blood agar plates were incubated at 37°C with 5% CO2 during 24 h for determinate viable cells.

### Phagocytosis Mediated by PSGL-1 Receptor

To analyze the function of the PSGL-1 receptor in recognizing the CPS of the new emerging serotypes 22F and 33F, and isogenic strains expressing different CPS, we followed the methodology previously described (Ramos-Sevillano et al., 2016). To block PSGL-1, HL-60 cells were incubated for 1 h at 37°C with 25 μg/ml of the KPL-1 antibody (mouse antihuman PSGL-1; MBL) or IgG isotype control (mouse anti-human IgG; Novus Biologicals). The phagocytosis killing assays were performed in the absence of rabbit serum using a proportion of 10^5^ HL-60 cells with the PSGL-1 receptor either active or blocked and 2.5 × 10^2^ CFU of *S. pneumoniae*. Then, as mentioned above, we plated on blood agar plates serial dilutions of culture for bacterial counts determination. Results were expressed normalizing CFU/ml to percentage of phagocytosis using the IgG isotype control as the 100%.

### Mouse Model of *S. pneumoniae* Sepsis

Experimental procedures involving mice were performed at Instituto de Salud Carlos III (ISCIII) conforming to the Spanish government legislation (RD 53/2013, ECC/566/2015) and European Community regulations (2010/63/EU). C57BL/6 male mice (8–12 weeks old) weighing about 20 g were bred by the ISCIII animal facility. Animal procedures followed the guidelines of the Bioethical and Animal Welfare Committee of ISCIII that reviewed and approved protocols (CBA PA 52-2011-v2 and PROEX 218/15). Studies investigating pneumococcal sepsis were performed using groups of five mice and infected as previously described (Ramos-Sevillano et al., 2016). Briefly, for sepsis, mice were challenged with 2 × 10^7^ CFU/mouse for P244, P017, 1407/18, 1000/18, 1833/18, and 1950/18 strains (Table 1) (in a volume of 200 μl) by the intraperitoneal route. Bacterial levels in blood, from the tail vein, were determined from every infected mouse at 24 h. The lower limit of detection was 10^2^ CFU/ml. Results were expressed as Log_10_ CFU/ml of bacteria recovered from the blood and survival curves were determined after 7 days of follow up.

### Statistical Analysis

Data represent results obtained from repeated independent experiments, each one conducted as a triplicate, representing at least three replicates. Statistical analysis was performed by using two-tailed Student’s *t*-test. The log-rank (Mantel-Cox) test was used for survival curves. Incidence rates were calculated considering the number of cases divided by the population using data provided by the Spanish National Institute of Statistics. Serotype trends were obtained by comparing the rates of the current situation (2017–2018) vs. the rates of different periods by calculating relative risks (IRR) with 95% confidence intervals through Poisson regression models. We included the pre-PCV13 period (2009), the early PCV13 effect (2010–2012) and the middle PCV13 period (2013–2016) when this vaccine was included in the pediatric calendar at national level at the end of 2016. Statistical analyses for epidemiological data were analyzed using Stata v.14. GraphPad InStat version 8.0 (GraphPad Software, San Diego, CA) was used for the rest of analysis. Differences were considered statistically significant with ^∗^*P* < 0.05 and highly significant with ^∗∗^*P* < 0.01 and ^∗∗∗^*P* < 0.001.

## Results

### Evolution of Invasive Pneumococcal Disease by Serotypes 22F and 33F in Spain

The epidemiological situation of IPD cases by clinical isolates of serotypes 22F and 33F was investigated during the last 10 years including 2009 which is the pre-PCV13 year and 2018 which is the last year when epidemiological data is fully registered in Spain.

The data contain cases, proportion among all IPD cases and incidences by these two serotypes. Incidences were calculated considering the number of cases divided by the population using data provided by the Spanish National Institute of Statistics. Incidence rate ratios with 95% confidence intervals were estimated to compare the current situation (2017–2018) vs. different periods including the pre-PCV13 period (2009), the early PCV13 effect (2010–2012) and the middle PCV13 period (2013–2016) when this vaccine was included in the pediatric calendar at national level at the end of 2016.

**FIGURE 1:**
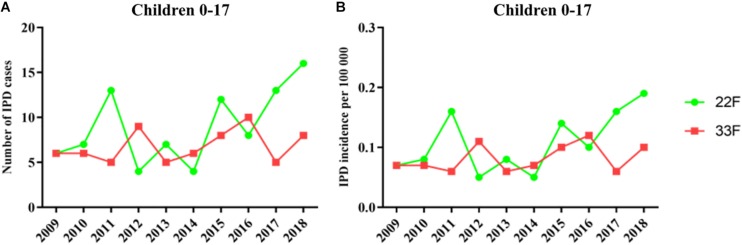
Trends in IPD incidence due to serotypes 22F and 33F producing IPD episodes in the pediatric population during the period 2009–2018 in Spain. **(A)** Number of IPD cases. **(B)** Incidence of IPD cases. Green line with dots is the evolution of serotype 22F whereas red line with squares is the evolution of serotype 33F.

In children, IPD cases by serotype 22F showed a similar trend until 2016 (6 cases in 2009 vs. 8 cases in 2016) which is the year when PCV13 was introduced at the national pediatric vaccination calendar (Figure 1A). However, since 2016 we observed a continuous rise with 13 cases in 2017 and 16 cases in 2018 or 29 cases in the last epidemiological period 2017–2018 showing the emerging of this serotype in children (Figure 1). The proportion of IPD cases by serotype 22F was 1.08% in 2009 and 6.23% in 2018 confirming that this serotype is rising and increasing its proportion over total serotypes in the pediatric population. IPD cases by serotype 33F in children show a moderate rise during the last 10 years when comparing 2009 vs. 2018 increasing from 6 cases in 2009 to 8 cases in 2018 and 13 cases for the last 2 years (Figure 1). Incidence rate ratios (IRR) for serotype 22F demonstrate an increasing trend (IRR 2.38; 95% CI 0.99–5.74 for the period 2017–2018 vs. 2009, IRR 1.82; 95% CI 1.06–3.12 for 2017–2018 vs. 2010–2012, and IRR 1.88; 95% CI 1.13–3.11 for 2017–2018 vs. 2013–2016 (Table 2). However, for serotype 33F the situation is relatively stable in the last 10 years (IRR 1.07; 95% CI 0.41–2.81 for the period 2017–2018 vs. 2009, IRR 0.98; 95% CI 0.49–1.97 for 2017–2018 vs. 2010–2012, IRR 0.90; 95% CI 0.47–1.73 for 2017–2018 vs. 2013–2016 (Table 2). Overall, our results suggest that serotype 22F is more prevalent in Spanish children than serotype 33F with a more pronounced effect in the last 2 years concurring with the introduction of PCV13 in the national immunization pediatric calendar.

**TABLE 2:** Number of cases and incidence of IPD in 2017–2018 compared to 2009 (pre-vaccine period), 2010–2012 (early effect after private use of PCV13 in Spanish children), and 2013–2016 (middle effect after PCV13 introduction).

		**2009 Pre-vaccine**		**2010–2012 Early vaccine effect (private market)**		**2013–2016 Middle vaccine effect**		**2017–2018**		**2017–18 vs. 2009**		**2017–18 vs. 2010–12**		**2017–18 vs. 2013–16**	
		**Cases**	**Incidence (per 100000)**	**Cases**	**Incidence (per 100000)**	**Cases**	**Incidence (per 100000)**	**Cases**	**Incidence (per 100000)**	**IRR**	**95% CI**	**IRR**	**95% CI**	**IRR**	**95% CI**
<18 years	22F	6	0.07	24	0.10	31	0.09	29	0.17	2.38	0.99–5.74	1.82	1.06–3.12	1.88	1.13–3.11
	33F	6	0.07	20	0.08	29	0.09	13	0.08	1.07	0.41–2.81	0.98	0.49–1.97	0.90	0.47–1.73
All adults	22F	64	0.17	271	0.23	364	0.24	248	0.32	1.95	1.48–2.56	1.39	1.17–1.65	1.37	1.16–1.61
	33F	26	0.07	72	0.06	125	0.08	88	0.11	1.70	1.10–2.64	1.86	1.36–2.54	1.41	1.15–1.74
18–64 years	22F	27	0.09	103	0.11	134	0.11	94	0.16	1.82	1.18–2.78	1.43	1.08–1.89	1.42	1.16–1.74
	33F	13	0.04	33	0.4	52	0.04	29	0.05	1.16	0.6–2.24	1.37	0.83–2.26	1.13	0.79–1.63
≥65 years	22F	37	0.48	168	0.69	230	0.68	154	0.87	1.83	1.28–2.62	1.26	1.01–1.57	1.29	1.1–1.51
	33F	13	0.17	39	0.16	73	0.21	59	0.33	2.00	1.1–3.64	2.08	1.39–3.11	1.56	1.21–2.01
Total IPD by all serotypes		3099	6.63	7009	4.95	8096	4.32	5243	5.62	0.85	0.81–0.89	1.13	1.09–1.18	1.30	1.25–1.34

*IRR = incidence rate ratio. CI = confidence interval.*

**FIGURE 2:**
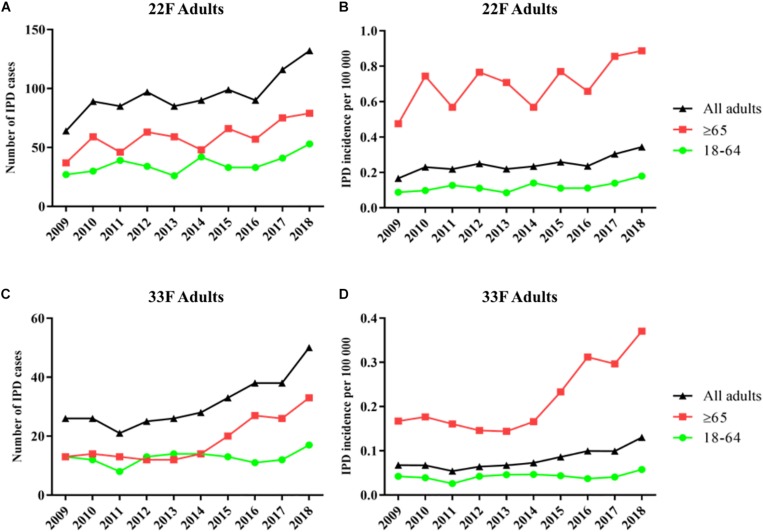
Trends in IPD incidence due to serotypes 22F and 33F producing IPD episodes in adults during the period 2009–2018 in Spain. **(A)** Number of IPD cases of serotype 22F. **(B)** Incidence of IPD cases of serotype 22F. **(C)** Number of IPD cases of serotype 33F. **(D)** Incidence of IPD cases of serotype 33F. Black line with triangles is the evolution in adults of all ages. Green line with dots is the evolution in adults 18–64 years. Red line with squares is the evolution in adults ≥65 years.

In adults of all ages, serotypes 22F and 33F show an increase of 52% (64 cases vs. 132 cases) and 48% (26 cases vs. 50 cases), respectively, when the pre-PCV13 period (year 2009) is compared to the last year 2018 (Figure 2). Hence, incidence by serotype 22F increased from 0.17 cases per 100000 in 2009 to 0.24 cases per 100000 in 2013–2016 and 0.32 cases per 100000 in 2017–2018. Incidence rate ratios show that serotype 22F rapidly increase after PCV13 was introduced in Spain with a constant increasing trend until 2018 (IRR 1.95; 95% CI 1.48–2.56 for 2017–2018 vs. 2009, IRR 1.39; 95% CI 1.17–1.65 for 2017–2018 vs. 2010–2012 and IRR 1.37; 95% CI 1.16–1.61 for 2017–2018 vs. 2013–2016) (Table 2). For serotype 33F, a similar pattern was observed after the introduction of PCV13 (IRR 1.70; 95% CI 1.10–2.64 for 2017–2018 vs. 2009, IRR 1.86; 95% CI 1.36–2.54 for 2017–2018 vs. 2010–2012 and IRR 1.41; 95% CI 1.15–1.74 for 2017–2018 vs. 2013–2016) (Table 2). In adults 18–64 years old, only serotype 22F increased since the introduction of PCV13 from 0.09 cases per 100000 in 2009 to 0.16 cases per 100000 in 2017–2018 (IRR 1.82; 95% CI 1.18–2.78). For adults ≥65 years, an increased in the incidence of both serotypes has been observed comparing 2009 and 2017–2018 (IRR 1.83; 95% CI 1.28–2.62 for serotype 22F and IRR 2.00; 95% CI 1.1–3.64 for serotype 33F). Hence, these data demonstrate that both serotypes 22F and 33F are emerging in adults being serotype 22F one of the most prevalent in 2018 for Spanish adults ≥65 years (3rd cause of IPD) whereas serotype 33F is the 16th cause of IPD in this age group (unpublished data from our laboratory).

### Genetic Analysis of Circulating Clones Among Clinical Isolates of Serotypes 22F and 33F

Genetic diversity of clinical isolates of serotypes 22F and 33F producing IPD in Spain was assessed by MLST. We chose the year 2018 to know the circulating genotypes of 22F and 33F because is an early time-point after the introduction of PCV13 in the pediatric calendar by the end of 2016 and even in the adult calendar of certain Spanish regions. In Spain, PCV7 was mainly used in private market and low selection pressure is expected.

Isolates of serotype 22F contained five different sequence types being ST433 the most frequent with up to 58% of the isolates belonging to this sequence type (Table 3). These genotypes were grouped into three different clonal complexes although CC433 was associated to 71% of the isolates of serotype 22F (Table 3). Isolates of serotype 33F also had five different genotypes being ST717 the most popular containing 71% of the strains, followed by ST 13320 with 18% of the isolates (Table 3). In contrast to serotype 22F, isolates of serotype 33F showed a less variable diversity in terms of clonal complexes with CC717 being the most frequent with up to 93% of the strains (Table 3). Overall, clinical isolates of serotypes 22F and 33F that are producing the majority of the IPD cases in the Spanish population seem to be very conserved with ST433 and ST717 being the most frequent genotypes among 22F and 33F, respectively (Table 3).

**TABLE 3:** Genotype and clonal complex distribution of *S. pneumoniae* 22F and 33F clinical isolates from the year 2018.

**Serotype/Clonal complex (CC)**	**Number of isolates**	**Percentage of total (%)**
Serotype 22F	24	
CC433	17	70,83
ST433	14	58,33
ST7314	3	12,5
CC698	5	20,83
ST698	2	8,33
ST13692	3	12,5
CC1439	2	8,33
ST3134	2	8,33
Serotype 33F	28	
CC717	26	92,86
ST717	20	71,43
ST13320	5	17,86
ST4668	1	3,57
CC1012	1	3,57
ST1012	1	3,57
CC433	1	3,57
ST7314	1	3,57

### Pneumococcal Biofilms of Serotypes 22F y 33F From Different Sources

**FIGURE 3:**
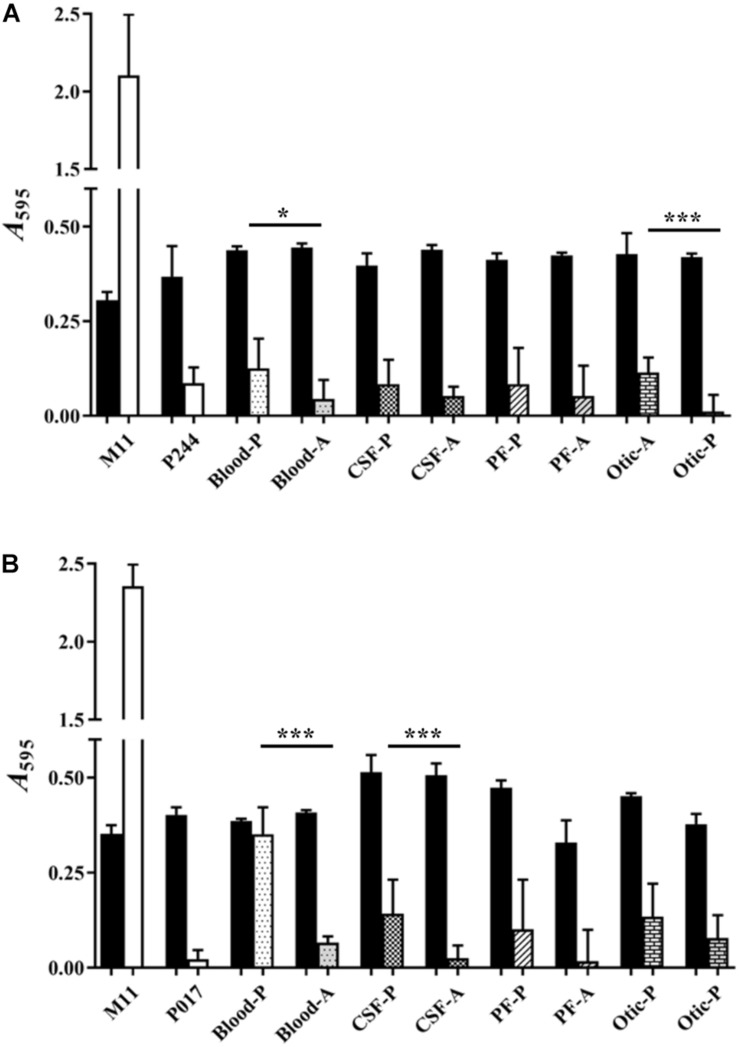
Biofilm formation of pediatric and adult clinical isolates from four different origins. **(A)** Represents clinical isolates of serotype 22F **(B).** Represents clinical isolates of serotypes 33F. M11, non-encapsulated strain; P244, 22F isogenic transformant; P017, 33F isogenic transformant; P, pediatric isolates; A, adult isolates; CSF, cerebrospinal fluid; PF, pleural fluid. Black bars represent growth and pattern or pattern-less bars represent biofilm formation. All data were normalized to the *A*_595_ CV blanks. Error bars represent standard deviations, and asterisks mark results that are statistically significant (two-tailed Student’s *t*-test: *P < 0.05; **P < 0.01; ***P < 0.001).

Figure 3 shows the biofilm formation capacity of clinical isolates of serotypes 22F (Figure 3A) and 33F (Figure 3B) from four different sources: blood, cerebrospinal fluid (CSF), pleural fluid (PF) and otic (Table 1). We also wanted to detect if there were differences in biofilm formation when the clinical isolate was either pediatric or adult (except for an adult clinical isolate of serotype 33F from otitis, which could not be tested). As biofilm formation controls, we used the non-encapsulated *S. pneumoniae* M11 strain, and isogenic mutants containing the genetic background of strain M11 and the capsules of serotypes 22F (P244) or 33F (P017). One clinical isolate of each serotype and origin were analyzed in detail. Although pneumococci of every serotype tested produced less biofilm than the non-encapsulated strain M11, significant differences were noted in the biofilm formation capacity of several pairs of clinical strains of the same serotype. The pediatric isolates formed greater biofilm than adult isolates, this difference being statistically significant in the case of clinical isolates from blood and otitis of serotype 22F (Figure 3A) and from blood and CSF of serotype 33F (Figure 3B). We also observed that there are clinical isolates of serotypes 22F or 33F that formed more or less biofilm than the isogenic transformant P244 or P017 (Figure 3). These results confirm previous observations indicating that the genetic background, and not only the CPS, modulates pneumococcal attachment to the artificial substrate (Moscoso et al., 2006; Domenech et al., 2014, 2015).

**FIGURE 4:**
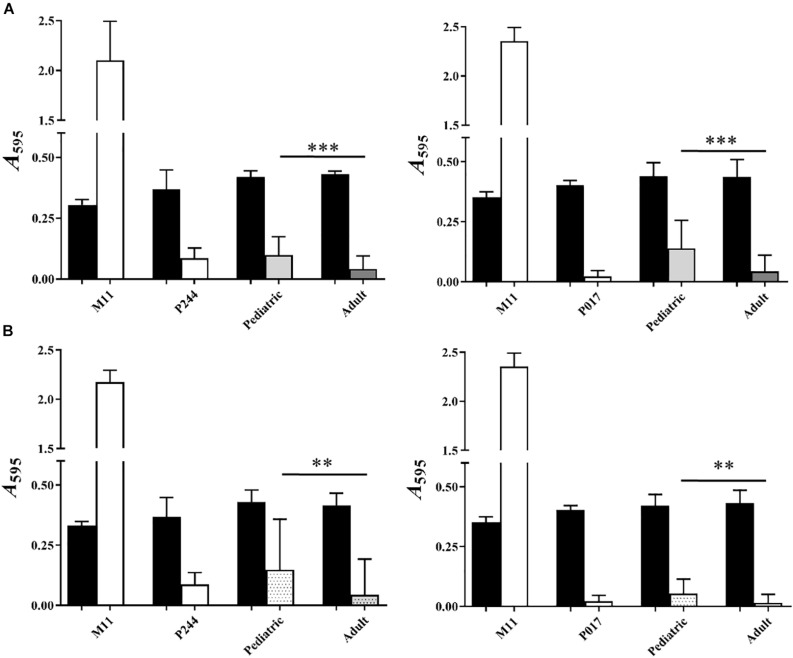
Analysis of biofilm formation of pediatric and adult clinical isolates. **(A)** Represents the comparison between adult and pediatric clinical isolates from four different origins: 22F (left) and 33F (right). **(B)** Represents the comparison between 8 additional adult and pediatric clinical isolates from blood: 22F (left) and 33F (right). M11, non-encapsulated strain; P244, 22F isogenic transformant; P017, 33F isogenic transformant. Black bars represent growth and white or color bars represent biofilm formation. All data were normalized to the *A*_595_ CV blanks. Error bars represent standard deviations, and asterisks mark results that are statistically significant (two-tailed Student’s *t*-test: ^∗^*P* < 0.05; ^∗∗^*P* < 0.01; ^∗∗∗^*P* < 0.001).

Comparing globally all the results of biofilm formation of pediatric isolates with those from adults (Figure 4A), biofilm formation was greater in clinical isolates of pediatric origin being statistically significant (^∗∗∗^*P* < 0.001). In Figure 3, we observed that, clinical pediatric isolates from blood were the best biofilm formers than isolates from the others sources (CSF, PF, otic) regardless of the serotype analyzed. In addition, if we compare a higher number of pediatric isolates from blood with adult isolates of each serotype (Figure 4B), we observed again, that pediatric blood isolates formed better biofilms than adult isolates and this phenotype was serotype-independent.

Comparison of ST and clonal complexes with biofilm formation based on the clinical origin of the isolates does not allow establishing a possible relationship between the genotype of the strain (Table 1 and Figures 3, 4), biofilm formation capacity and the origin of the sample.

### Opsonophagocytosis of Different Serotypes With the Same Genetic Background Growing as Planktonic or Biofilms Cultures

**FIGURE 5:**
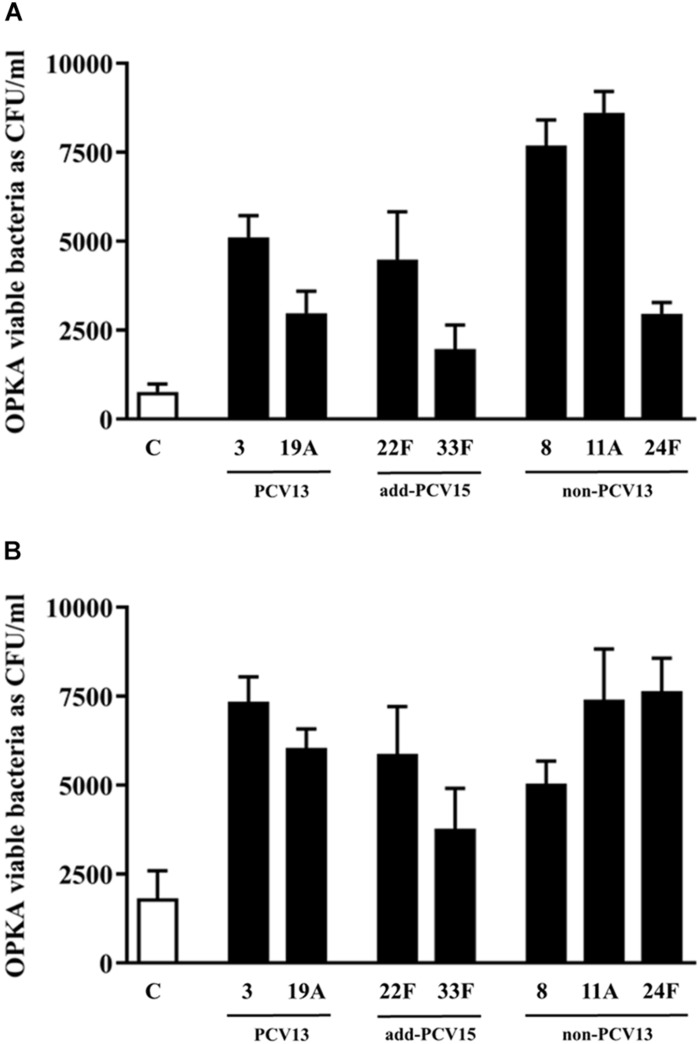
Influence of different CPS in the evasion of opsonophagocytosis. **(A)** Bacterial survival of control strain M11 expressing different CPS and grown as a planktonic culture. **(B)** Bacterial survival of control strain M11 expressing different CPS and grown as a biofilm. Error bars represent standard deviations, and asterisks mark results that are statistically significant (two-tailed Student’s *t*-test: *P < 0.05; **P < 0.01; ***P < 0.001).

To explore the potential of different serotypes to avoid the opsonophagocytosis process by human neutrophils which could explain differences in the burden of disease at the epidemiological level, isogenic transformants of M11 strain were used (Figure 5). For this study, PCV13 serotypes such as 3 and 19A and non-PCV13/PCV15 serotypes such as 8, 11A and 24F were included based in their high prevalence rates of these serotypes causing IPD in Europe (Fenoll et al., 2015). Serotype 11A is an emerging multidrug resistant phenotype and a good biofilm former (Aguinagalde et al., 2015; Domenech et al., 2015), whereas serotypes 22F and 33F contained in PCV15 were included for comparison purposes.

The use of the M11 strain for constructing all the isogenic transformants, being the CPS as the only difference among the strains, is the best strategy to assess variations in the phagocytosis process mediated by the CPS and not by differences related to the genetic background of the strains. OPKA was measured using bacteria growing as planktonic culture or biofilms.

In planktonic cultures, the analysis of the two most frequent PCV13 serotypes, confirmed that isolate with CPS of type 3 was more resistant to the phagocytosis than the isolate expressing serotype 19A proving than the CPS of serotype 3 conferred more resistance to the phagocytosis than the capsule of type 19A (^∗∗∗^*P* < 0.001) (Figure 5A). The two new additional serotypes included in PCV15 showed a different pattern of recognition by the phagocytic cells. The capsule 22F was more resistant to the phagocytosis process than the capsule 33F (^∗∗∗^*P* < 0.001) (Figure 5A). These results may explain why serotype 22F is more frequent causing IPD in children and adults than serotype 33F (Figures 1, 2). Strains expressing the CPS of types 8 and 11A had the most resistant phenotype whereas the strain with the CPS of type 24F was phagocytosed as the same level than the CPS of 19A (Figure 5A).

In biofilm cultures, the strain expressing serotype 3 was more resistant to the phagocytosis process than serotype 19A upholding the marked resistant level of serotype 3 to the phagocytosis (Figure 5B, ^∗∗∗^*P* < 0.001). Among the two additional serotypes included in PCV15, the strain expressing the CPS of serotype 22F was more resistant to phagocytosis than the strain of serotype 33F suggesting that serotype 22F may have an enhanced ability to colonize and produce persistent infection associated to biofilms than serotype 33F (Figure 5B). The strain with the CPS of type 8 had a similar pattern of phagocytosis evasion than the strain expressing the serotype 19A (Figure 5B). Finally, serotypes 11A and 24F avoided very efficiently the phagocytosis in comparison to the control strain (Figure 5B).

### Evasion of Phagocytosis by Clinical Isolates of Serotypes 22F and 33F From Children and Adults

**FIGURE 6:**
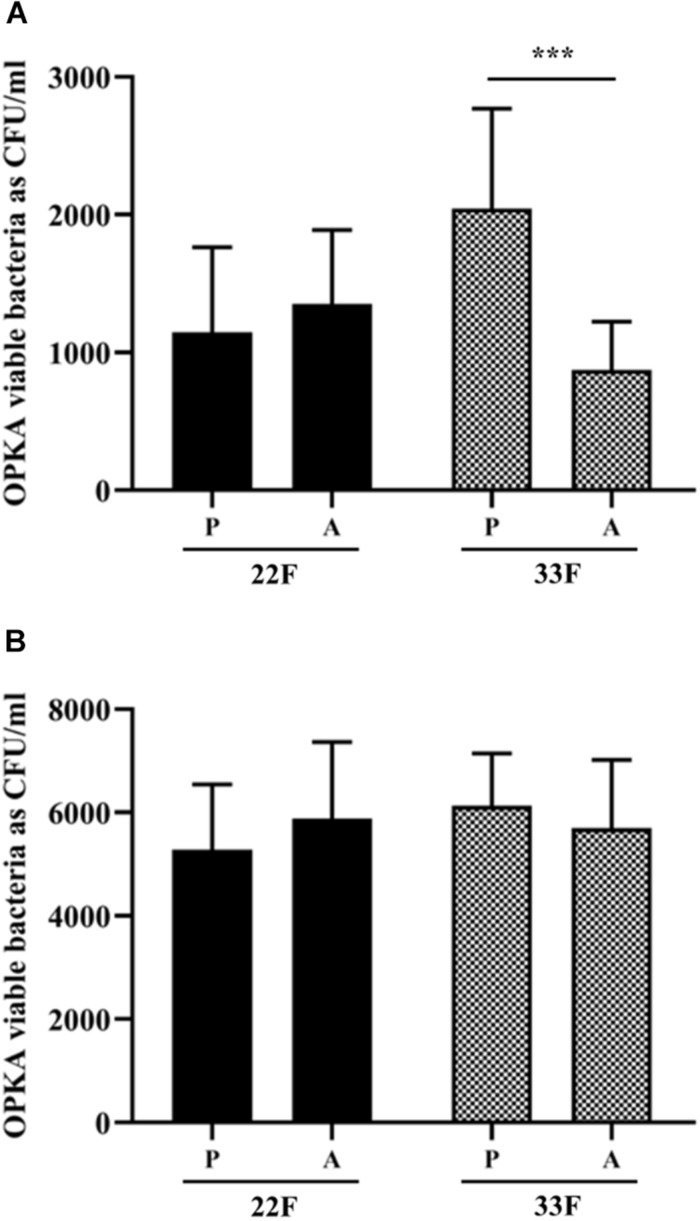
Evasion of opsonophagocytosis of 22F and 33F clinical isolates. **(A)** Bacterial survival of clinical strains from pediatric (P) and adult (A) patients and grown as a planktonic culture. **(B)** Bacterial survival of clinical strains from pediatric (P) and adult (A) patients and grown as a biofilm. Error bars represent standard deviations, and asterisks mark results that are statistically significant (two-tailed Student’s *t*-test: ***P < 0.001).

In this study, we analyzed the recognition of pneumococcal serotypes 22F and 33F by phagocytic cells using clinical isolates from blood obtained from children and adults with IPD and grown as planktonic or biofilms (Figure 6). For serotype 22F, no differences were found between pediatric or adult isolates suggesting that this serotype may affect both target populations at a similar level and therefore, it may be useful to vaccinate children and adults against serotype 22F (Figure 6). In the case of serotype 33F, a higher resistance level to phagocytosis was observed in the pediatric isolate grown as planktonic culture (Figure 6A) whereas no differences were observed between isolates as biofilms (Figure 6B), suggesting that children may be more susceptible to IPD by this serotype.

### Role of PSGL-1 Receptor in the Phagocytosis of Prevalent Pneumococcal Serotypes Included or Not in Conjugate Vaccines

**FIGURE 7:**
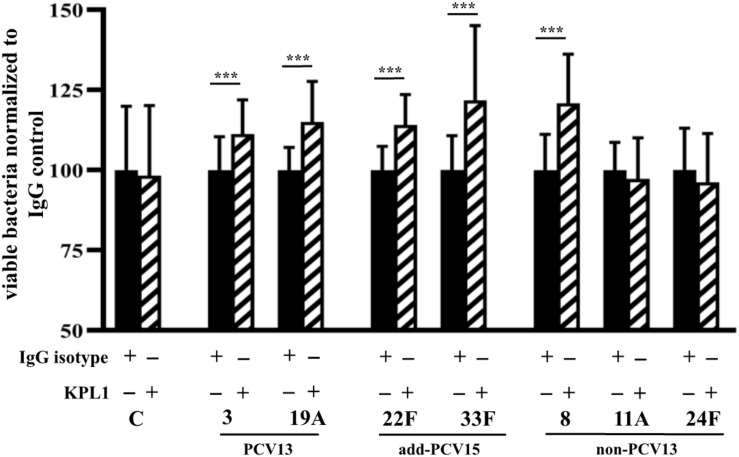
Phagocytosis mediated by PSGL-1 receptor. Results represent bacterial survival of control strain M11 expressing different CPS and grown as a planktonic culture. Phagocytic cells were incubated with an IgG isotype negative control that does not block PSGL-1 (black bars), whereas cells exposed to the KPL-1 antibody had the PSGL-1 blocked (hatched bars). Error bars represent standard deviations, and asterisks mark results that are statistically significant (two-tailed Student’s *t*-test: ***P < 0.001).

The interaction of pneumococcal serotypes with the PSGL-1 receptor was studied in the absence of serum to avoid any effect on phagocytosis by serum-mediated receptors such as complement receptors. In the absence of pneumococcal capsule (control strain M11), we did not observe any difference on phagocytosis independently of the functionality of the PSGL-1 receptor, confirming that the capsule is necessary for the PSGL-1 mediated phagocytosis (Figure 7). For serotypes 3 and 19A, blocking of PSGL-1 by treatment with KPL1 antibody, increased the viability of bacterial cells, confirming that PSGL-1 recognizes pneumococcal CPS of types 3 and 19A inducing phagocytosis (Figure 7). Similar results were obtained for the additional serotypes included in PCV15 and for serotype 8 as inhibition of PSGL-1 receptor on human neutrophils by KPL1 allowed greater levels of viable bacteria of these serotypes, demonstrating that this receptor is important for the phagocytosis of serotypes 22F, 33F and 8 (Figure 7). However, uptake of serotypes 11A and 24F remained similar in the presence or absence of PSGL-1 (Figure 7) suggesting that these two serotypes are not recognized by this receptor which may explain the emergence of these two serotypes causing IPD cases in the last years.

### Virulence of Isolates of Serotypes 22Fand 33F in a Mouse Sepsis Model of Infection

**FIGURE 8:**
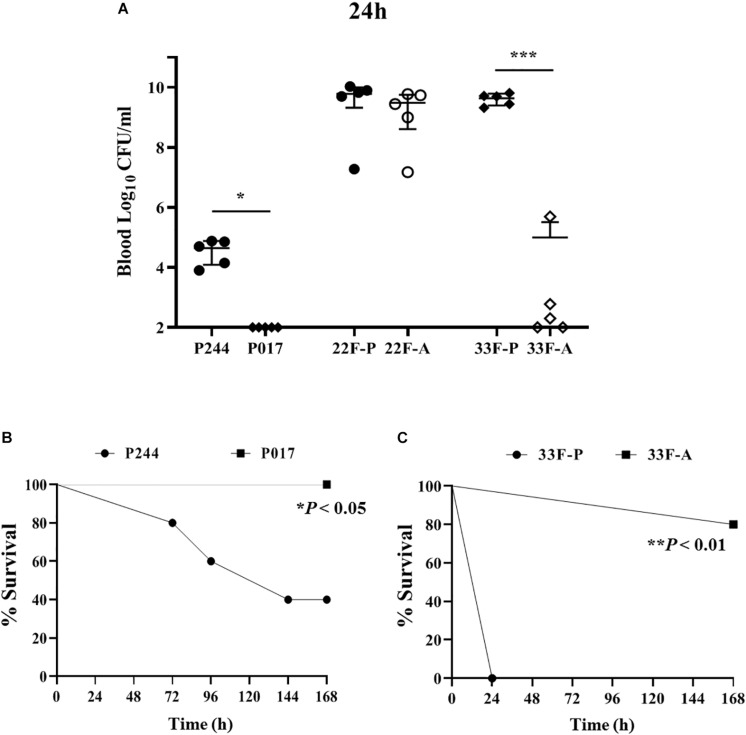
Virulence of isolates of serotypes 22F and 33F in a sepsis model of infection. **(A)** Colony counts expressed as Log_10_ CFU/ml from the blood of mice infected with M11 strain expressing serotype 22F (P244) or serotype 33F (P017) or with clinical isolates of serotypes 22F or 33F from pediatric origin (P) or adult origin (A). **(B)** Survival curves of P244 vs. P017 strains. **(C)** Survival curves of clinical isolates of serotype 33F from pediatric origin (P) or adult (A). Error bars represent standard deviations, and asterisks mark results that are statistically significant (two-tailed Student’s *t*-test: *P < 0.05; ***P < 0.001 for bacterial counts; and Log-rank test *P < 0.05; **P < 0.01 for survival curves).

Infection assays using the isogenic transformants of M11 strain expressing the CPS of type 22F (P244) or 33F (P017) confirmed that the CPS of serotype 22F confers a higher invasive capacity than the CPS of serotype 33F as in these two strains the genetic background is the same and the only difference is the CPS expressed (Figure 8). The M11 strain expressing 22F had a higher ability to replicate in blood (Figure 8A) and lethality was increased compared to M11 with CPS of type 33F (Figure 8B). This finding is consistent with the epidemiological results mentioned above showing that serotype 22F is more prevalent than serotype 33F in IPD in Spain. When clinical isolates of both serotypes were analyzed, we found that serotype 22F is very lethal regardless of the origin of the strain, killing all the mice within the first 30 h (Figure 8). However, in serotype 33F, the replication in blood of the pediatric isolate was markedly higher compared to the adult isolate (Figure 8A) showing a marked lethality (Figure 8C) which explains the higher resistance to the phagocytosis of this strain in the planktonic cultures (Figure 6A).

## Discussion

The effectiveness of current pneumococcal conjugate vaccines against targeted serotypes has been very successful decreasing the burden of disease caused by these serotypes, although unfortunately some countries have reported an increase of IPD cases by certain non-vaccine types in recent years (Feikin et al., 2013; Savulescu et al., 2017; Ladhani et al., 2018; Lewnard and Hanage, 2019). This is the case for serotypes 22F and 33F with different countries reporting increased rates in children and adults after PCV13 (Kaplan et al., 2013; Pichon et al., 2013; Moore et al., 2015; Golden et al., 2016; Balsells et al., 2017; Cui et al., 2017). Serotypes 22F and 33F are also important in terms of quality adjusted life years (QALY) as they caused the highest QALY loss among non-PCV13 serotypes in children (van Hoek et al., 2012). In Spain, our data show an increase of IPD cases for serotype 22F in children and adults and a moderate increase for serotype 33F mainly in adults which is consistent with the situation of other countries worldwide described above. Interestingly, serotype 22F significantly increased not only in adults up to 64 years old but also in adults ≥65 years old between 2009 and the 2017–2018 period (IRR 1.82; 95% CI 1.18–2.78 and IRR 1.83; 95% CI 1.28–2.62, respectively), whereas serotype 33F only increased significantly in the population ≥65 years (IRR 2.00; 95% CI 1.1–3.64). The higher trend to infect adults regardless the age in the case of serotype 22F may explain why this serotype is increasing at a higher proportion than serotype 33F in other countries (Golden et al., 2016; Ladhani et al., 2018). The clonal spread of pneumococcal population allows the dissemination of hypervirulent clones and multidrug resistant genotypes that may contribute to the expansion of certain serotypes and genotypes (Brueggemann and Spratt, 2003; Ardanuy et al., 2014; Aguinagalde et al., 2015; Azarian et al., 2018). Our data demonstrate that genotype ST433 is the most frequent cause of IPD among clinical isolates of serotype 22F in Spain. This is consistent with results from other countries for this serotype causing IPD (Golden et al., 2016) although it also has been found associated to nasopharyngeal carriage (Sa-Leao et al., 2011). For serotype 33F, our results show up to five different genotypes whereas isolates of this serotype from other countries show a more clonal diversity with up to eight different genotypes (Golden et al., 2018). In addition, the majority of the clinical isolates of serotype 33F that are circulating in Spain, belonged to the genotype ST717, which is different to the situation in other countries such as Canada where ST100 is the most frequent cause of IPD for serotype 33F (Golden et al., 2016).

The use of isogenic transformants to make comparisons of biofilm formation is very useful to avoid differences of each clinical isolate (Domenech et al., 2014, 2015), since biofilm formation is a multifactorial process (Domenech et al., 2013). Hence, to analyze the influence of the CPS, the most reasonable thing would be to use bacterial lines with the same genetic background. Biofilm formation is a tool that can help to predict the clinical impact and emergence of new serotypes that colonize the nasopharynx after the introduction of new vaccines (Domenech et al., 2014). These authors observed that serotype 19A was an excellent biofilm former with a marked capacity of nasopharyngeal colonization (Domenech et al., 2014). This ability to form thick biofilms can contribute to the pathogenesis process of serotype 19A, explaining why is one of the most predominant PCV13 serotypes in IPD (Domenech et al., 2014; Fenoll et al., 2015; Ladhani et al., 2018). A similar approach can be useful to explain the emergence of serotype 11A because is also a good biofilm former and is associated to multidrug resistance (Aguinagalde et al., 2015; Domenech et al., 2015). Serotypes 8 and 3, despite forming little biofilm, remain two of the most important serotypes in IPD, with serotype 8 being the most predominant in Europe and the second in Spain, which shows that biofilm formation is not the only factor to take into account when determining the clinical impact of serotypes and their future impact on the incidence of the disease. *S. pneumoniae* isolates of serotypes 22F and 33F, which were found both in carriage and causing IPD, and which are potentially highly invasive (Yildirim et al., 2010), failed to produce substantial amounts of biofilms *in vitro*. We observed the formation of pneumococcal biofilm for clinical strains expressing capsules of types 22F or 33F although in the majority of the cases it was weak in agreement with previous observations using isogenic transformants with CPS 22F and 33F (Domenech et al., 2015). The biofilm levels of the control strain M11 in our study are lower than those found in the study by Domenech et al., mentioned above, but this aspect does not affect the findings of the manuscript as we used the same M11 strain to construct the isogenic mutants. The use of different brands of multiwell plates, cristal violet, and a different multiplate reader may have contributed to the variations of absorbance values. In our study, the formation of biofilm does not explain the increase in IPD by serotypes 22F and 33F, since being bad biofilm formers, an expected phenotype that was already described in previous work (Domenech et al., 2015), they should not be very frequent in IPD. However, considering that colonization of the nasopharynx is a multifactorial process and that although the capsule is a prerequisite for the virulence of the microorganism, there are numerous virulence factors that also contribute to the pathogenesis of IPD and for serotypes 22F and 33F may be playing a predominant role (Geno et al., 2015). The CPS, determines the pneumococcal surface charge, and this aspect influences colonization, because negatively charged surfaces are associated with increased colonization (Weinberger et al., 2009). Most pneumococcal capsules are anionic and most pneumococcal clinical isolates have negative charge (Geno et al., 2015; Morais et al., 2018). This anionic charge helps preventing clearance by the mucus and helps repelling phagocytosis. There are many examples of capsule-negative strains that colonize the nasopharynx and also cause disease, such as serotype 19A, which is a good biofilm former *in vitro* (Domenech et al., 2014), colonizes efficiently the human nasopharynx (Adler et al., 2019) and is one of the main PCV13 serotypes causing IPD. In addition, anionic charge of the capsule does not seem to be an impediment to colonize the upper respiratory tract and produce IPD, but there are capsule-negative serotypes that are good biofilm formers such as 35B and capsule-negative serotypes that are bad biofilm formers such as 24F (Domenech et al., 2015). This phenomenon would explain that serotype 22F is more prevalent in the population, since due to the structure of its CPS its net charge is negative whereas the CPS of 33F is neutral (Geno et al., 2015). These differences in the CPS charge on 22F could contribute to a better colonizing phenotype, and could explain why there is a greater burden of disease due to serotype 22F compared to 33F.

The heterogeneity in biofilm formation using clinical isolates of this study is not surprising, since it confirms evidence published before, where the genetic background and not only the CPS modulate the adhesion of the pneumococcus and formation of a biofilm on an artificial substrate (Moscoso et al., 2006; Domenech et al., 2009, 2014). The novelty of this work, resides in the differences found between pediatric biofilms and adult biofilms, more precisely, the tendency of pediatric isolates to form greater biofilms. These results are compatible with the fact that the pediatric population is the largest carrier of pneumococcus with up to 20–40% of asymptomatic colonization in healthy children (Chaguza et al., 2019) suggesting that pneumococcal isolates can modulate its ability to interact with the nasopharyngeal epithelium by a mechanism related to increased activation of intrinsic factors associated with biofilm formation. The source of the strain (pediatric or adult) is certainly not the only difference between the phenotype of the strains and although the genotype did not contribute to our results, other unrelated factors may be affecting.

Resolution of IPD is finely regulated by the efficient recognition and clearance of *S. pneumoniae* by professional phagocytes (Standish and Weiser, 2009; van der Poll and Opal, 2009). The pneumococcal CPS that surrounds the bacterium and it is used to determine the serotype, is the most well-established virulence factor, although the genetic background also contributes significantly to the pathogenesis process (Hyams et al., 2011; McAllister et al., 2011). Our study using strains with the same genetic background but expressing different CPS, demonstrates that the strain with capsule of type 3 was more resistant to the phagocytosis process that the strain expressing the capsule 19A. This is interesting because serotype 3 remains as one of the most frequent causes of IPD in adults worldwide (Moore et al., 2015; Ladhani et al., 2018). The higher resistant phenotype conferred by the capsule of type 3 may be due to the higher size and even abundance of the CPS expressed on these strains (Choi et al., 2015). Among the two additional serotypes covered by PCV15, the strain expressing the CPS of type 22F was more resistant to the phagocytosis and much more virulent in a mouse sepsis model than the strain of serotype 33F. This is consistent with the epidemiological data presented in our study showing a higher burden of disease caused by serotype 22F and may explain why in other countries, serotype 22F is also more prevalent than serotype 33F (Moore et al., 2015; Golden et al., 2016; Ladhani et al., 2018). In bacteria growing as biofilms, strains expressing the CPS of type 3, 11A and 24F were among the most resistant strains to be phagocytosed. This is interesting as serotype 3 remains as major cause of pneumonia in adults (Menéndez et al., 2017) and in patients with COPD, serotypes 3 and 11A are among the most prevalent (Shoji et al., 2018). In addition, serotype 24F is more prone to cause meningitis, showing an increase in clinical severity (Balsells et al., 2018) which could be associated to the increase resistant pattern to phagocytosis when replicating as a biofilm.

The repertoire of cellular receptors involved in the phagocytosis of invading pathogens is critical for the outcome of the infection. PSGL-1 on leukocytes plays a critical role in host defense against pneumococcal infection. As a consequence of pathogen-recognition by PSGL-1, pneumococcal strains are efficiently phagocytosed and killed intracellularly, reducing bacterial replication and dissemination in the host, contributing therefore, to control the severity of the IPD process (Ramos-Sevillano et al., 2016). Our results show that PSGL-1 receptor is important for the phagocytosis of different serotypes including the additional serotypes covered by PCV15. However, for serotypes 11A and 24F, cells expressing PSGL-1 did not kill efficiently these particular serotypes suggesting that strains expressing these two CPS, may have an increased ability to produce IPD. This is consistent with the emergence of amoxicillin-resistant variants of serotype 11A with genotype ST6521 that avoid efficiently the phagocytosis process and are good biofilm formers (Aguinagalde et al., 2015) and with the increased severity of infection by isolates of serotype 24F (Balsells et al., 2018).

Overall, the results of this study may be of relevance to understand critical aspects of the pathogenesis process such as biofilm formation and interaction with phagocytes by prevalent serotypes including the additional serotypes covered by the new PCV15. Surveillance studies and characterization of emergent pneumococcal strains remains necessary in order to explain what features lead to the success of individual serotypes and genotypes.

## Data Availability Statement

The datasets generated for this study are available on request to the corresponding author.

## Ethics Statement

Animal procedures followed the guidelines of the Bioethical and Animal Welfare Committee of ISCIII that reviewed and approved protocols (CBA PA 52-2011-v2 and PROEX 218/15) and were performed conforming to the Spanish Government legislation (RD 53/2013, ECC/566/2015) and European Community regulations (2010/63/EU).

## Author Contributions

JS, MD, and JY conceived and designed the experiments and wrote the manuscript. SM carried out the epidemiology analyses. JS and MD performed the experiments. FG-C helped with the interpretation of the data. SM and FG-C reviewed the results and contributed to the final version of the manuscript.

## Conflict of Interest

JY has received a grant from MSD-USA (MISP Call) and has received personal fees from the GSK, MSD, and Pfizer. The remaining authors declare that the research was conducted in the absence of any commercial or financial relationships that could be construed as a potential conflict of interest.

## Footnotes

1 http://pubmlst.org/spneumoniae
